# Oral formulation of bendamustine hydrochloride for patients with advanced solid tumors; a phase 1 study

**DOI:** 10.1007/s10637-022-01307-6

**Published:** 2022-11-04

**Authors:** Toshio Shimizu, Kazuhiko Nakagawa, Hidetoshi Hayashi, Tsutomu Iwasa, Hisato Kawakami, Satomi Watanabe, Noboru Yamamoto, Kan Yonemori, Takafumi Koyama, Jun Sato, Kenji Tamura, Keiichi Kikuchi, Kenichiro Akaike, Shiho Takeda, Masayuki Takeda

**Affiliations:** 1 Department of Experimental Therapeutics, National Cancer Center Hospital, 5-1-1 Tsukiji Chuo-ku Tokyo, Japan; 2 Department of Pulmonary Medicine and Medical Oncology, Wakayama Medical University Graduate School of Medicine, Wakayama Medical University Hospital, 811-1 Kimiidera, Wakayama-City, Wakayama, Japan; 3 Department of Medical Oncology, Kindai University Faculty of Medicine, 377-2, Osakasayama-city, Osaka, Japan; 4 Innovative Cancer Center / Department of Medical Oncology, Faculty of Medicine, Shimane University, 89-1. Enyacho Izumo-city, Shimane, Japan; 5 SymBio Pharmaceuticals Limited, 3-2-2 Toranomon, Minato-ku, Tokyo, Japan; 6 Department of Cancer Genomics and Medical Oncology, Nara Medical University, 840 Shijo-Cho, Kashihara, Nara, Japan

**Keywords:** Bendamustine, Oral formulation, Pharmacokinetics, Phase I study, Solid tumor

## Abstract

To determine the maximum tolerated dose (MTD) and recommended dose (RD) of orally-administered bendamustine in Japanese patients with advanced solid tumors. The optimal dosing schedule, safety, pharmacokinetics, and preliminary antitumor effects were also evaluated. A multicenter, open-label trial with a standard 3 + 3 design and dose escalation by dose-limiting toxicity (DLT) was conducted. The treatment schedules were once daily for 7, 14, and 21 days every 3 weeks as one cycle. The total dose per cycle was increased from 175 to 840 mg/m^2^. Eighteen patients were enrolled in this study. DLT occurred in one of six patients at 75 mg/m^2^/day × 7 days, and one of three patients at 37.5 mg/m^2^/day × 14 days and 25 mg/m^2^/day × 21 days. However, the delayed recovery from a decrease in neutrophil or platelet count hampered the start of subsequent treatment cycles, and the trend was more prominent at 37.5 mg/m^2^/day × 14 days and 25 mg/m^2^/day × 21 days than in 75 mg/m^2^/day × 7 days. MTD was determined as 75 mg/m^2^/day × 7 days to allow acceptable hematologic recovery. The pharmacokinetics of orally-administered bendamustine were generally dose-dependent; however, the inter-individual variability is relatively large. The major adverse events were hematologic toxicities; gastrointestinal disorders were generally mild. Adverse drug reactions did not lead to the discontinuation of the drug. A partial response was observed in two of six patients (prostatic small cell carcinoma and thymic carcinoma) at 75 mg/m^2^/day × 7 days. The RD and optimal dosing schedule of orally-administered bendamustine was 75 mg/m^2^ once daily for 7 days every 3 weeks for the treatment of advanced solid tumors. (*Trial registration number* ClinicalTrials.gov NCT03604679. Registration date July 27, 2018).

**Supplementary Information:**

The online version contains supplementary material available at 10.1007/s10637-022-01307-6.

## Introduction

Drug therapies for malignant tumors include chemotherapy, hormone therapy, and molecular-targeted therapy, and a combination of these therapies has been used in clinical settings. Chemotherapy has long been used to treat cancer and remains the backbone of systemic treatment in most cancers. Bendamustine hydrochloride (bendamustine) is a less toxic alkylating agent composed of three structures: a nitrogen mustard skeleton, benzimidazole ring, and butyric acid side chain. It was first synthesized in the German Democratic Republic in 1963 [1]. Bendamustine has achieved widespread international regulatory approval and is a standard agent for the treatment of indolent non-Hodgkin lymphoma (NHL), chronic lymphocytic leukemia (CLL), and multiple myeloma (MM) [2].

The alkylation activity of bendamustine was found to be more potent than that of other alkylating agents in previous studies [3–5]. Because it possesses a purine skeleton, it also has an anti-DNA metabolic effect [6]. Clinical studies using injectable bendamustine have demonstrated high response rates with monotherapy in indolent NHL and CLL [7, 8] and have confirmed its efficacy in MM [9]. In Japan, bendamustine injection has been approved for the treatment of indolent B-cell NHL, mantle cell lymphoma (MCL), CLL, and lymphodepletion in tumor-specific chimeric antigen receptor T-cell (CAR-T) therapy. Recently, bendamustine injection was approved for the treatment of relapsed or refractory diffuse large B-cell lymphoma in 2021.

Bendamustine has also been extensively evaluated for the treatment of solid tumors, and its activity has been observed in patients with metastatic breast cancer (mBC) and small cell lung cancer (SCLC) [10]. Single-agent bendamustine has shown promise in the treatment of relapsed mBC [11, 12], and the superiority of bendamustine, methotrexate, and 5-fluorouracil over cyclophosphamide, methotrexate, and 5-fluorouracil has been demonstrated in a phase 3 trial for the first-line treatment of mBC [13]. Similarly, single-agent bendamustine is also active in the treatment of relapsed SCLC [14, 15], and promising efficacy has been reported in combination with carboplatin for the first-line treatment of extensive stage SCLC [16]. The advantage of the bendamustine combination was that the non-hematologic toxicity rates were very low, and the regimen was not contraindicated in patients with cardiovascular disease.

Oral administration of bendamustine to humans was first reported by Preiss et al. in 1985, and a moderate bioavailability of 57% was reported [17]. Oral drug administration is the most suitable route for the treatment of solid tumors in outpatient settings; however, an oral formulation of bendamustine has not been developed to date. Therefore, a liquid-filled hard capsule (LFHC, orally-administered bendamustine), containing emulsified bendamustine in a non-aqueous mixture, was developed as an oral formulation, to improve the quality of life (QOL) and convenience of patients and its expansion to indications of solid tumors was investigated.

The development of a new oral dosing schedule for orally-administered bendamustine that ensures a balance between efficacy and safety will provide a new treatment option for patients with advanced solid tumors. In this study, we employed a standard 3 + 3 dose escalation design to evaluate the safety and pharmacokinetics (PK) and to determine the recommended dose (RD) and optimal dosing schedule of orally-administered bendamustine in patients with advanced solid tumors. Furthermore, we explored the types of tumors that were sensitive to orally-administered bendamustine.

## Materials and methods

### Study design

**Fig. 1:**
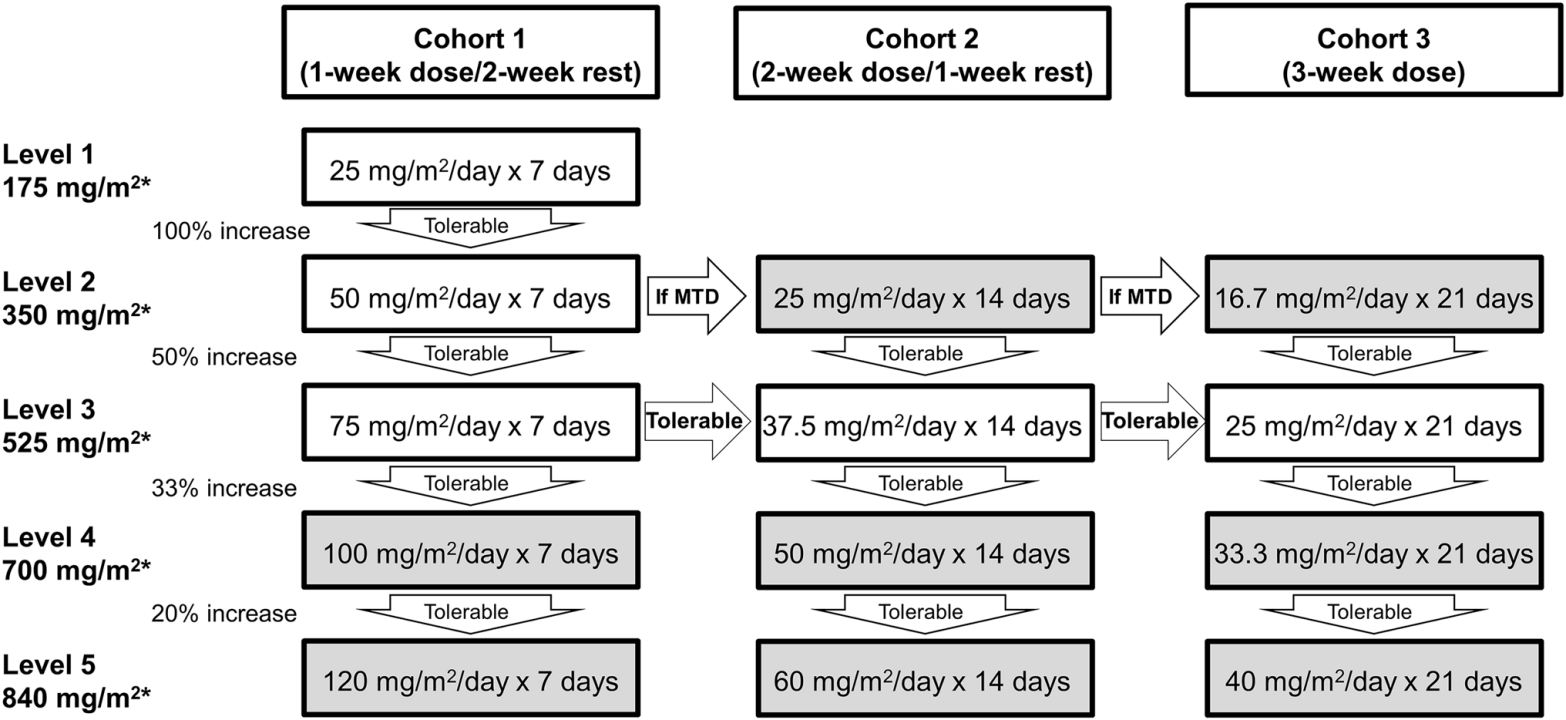
Dose escalation schedule of orally-administered bendamustine per cohort and level. Thick frame indicates the evaluated dosage, and gray highlight indicates the unexamined dosage at each level in each cohort; MTD, maximum tolerated dose. *Total dose of bendamustine per cycle (3-week schedule)

This study (NCT03604679) was conducted from May 16, 2018 to May 12, 2020 in Japan as a two-center, open-label, standard 3 + 3 dose escalation study, according to Good Clinical Practice, the Declaration of Helsinki, and other applicable regulations. A 3-week treatment period was considered as one cycle, and the maximum tolerated dose (MTD) and RD were assessed in Cohort 1 (once daily for 7 days), 2 (once daily for 14 days), and 3 (once daily for 21 days). Patient enrollment began with Cohort 1-Level 1 and was increased using a 3 + 3 design based on the dose-limiting toxicity (DLT). The starting dose was determined to be 25 mg/m^2^/day for cohort 1, based on the overall area under the concentration–time curve (AUC) expected for orally-administered bendamustine. The absolute bioavailability of the bendamustine LFHC formulation was evaluated in patients with hematologic malignancies and reported as 66% after administration in a fasted state [data on file]. Therefore, the overall AUC of orally-administered bendamustine 25 mg/m^2^/day (total175 mg/m^2^ pre cycle as Cohort 1-Level 1) was expected to be approximately half of the maximum approved dose for bendamustine injection (120 mg/m^2^ daily for two consecutive days, every 21 days cycle, overall 240 mg/m^2^/cycle). Thereafter, the orally-administered bendamustine dose was escalated through Levels 2, 3, 4, and 5 at 350, 525, 700, and 840 mg/m^2^/cycle, respectively (Fig. 1), where the equivalent dose to bendamustine injection was 350 mg/m^2^/cycle.

Three to six patients were enrolled at each level in each cohort, starting with Cohort 1-Level 1 (25 mg/m^2^/day × 7 days). If Cohort 1-Level 1 reached the MTD, Cohort 2 and 3 were not assessed. If Cohort 1-Level 2 (50 mg/m^2^/day × 7 days) reached the MTD, Cohort 2-Level 2 (25 mg/m^2^/day × 14 days) was initiated. If Cohort 1-Level 3 (75 mg/m^2^/day × 7 days) was tolerated, Cohort 2-Level 3 (37.5 mg/m^2^/day × 14 days) and Cohort 3-Level 3 (25 mg/m^2^/day × 21 days) were started simultaneously.

The daily doses at each level in each cohort are shown in Fig. 1. When non-hematologic toxicity greater than grade 3 or hematologic toxicity (neutropenia or thrombocytopenia greater than grade 4 or febrile neutropenia (FN) greater than grade 3) was observed, the dose was interrupted and restarted after recovery with a reduced dose to the previous level in the same cohort. Dose reduction was allowed twice, and treatment was discontinued if further dose reduction was required at the lowest level in the cohort. Treatment continuation was allowed until the criteria for discontinuation/termination were met, without any limit on the number of cycles.

DLT was defined as the following adverse events (AEs) for which a causal relationship to the study drug could not be ruled out: 1) Grade 4 neutrophil count decrease, lasting for more than 8 days; 2) FN; 3) grade ≥ 3 platelet count decrease, requiring platelet transfusion; 4) grade ≥ 3 anemia requiring red blood cell transfusion; 5) Grade 4 platelet count decrease; 6) Grade ≥ 3 non-hematologic toxicity except in grade ≥ 3 nausea, vomiting, and diarrhea controlled with appropriate supportive care or grade ≥ 3 abnormalities in laboratory test results controlled with appropriate supportive care or without clinically significant symptoms; and 7) unresolved hematologic toxicity (platelet count < 75,000/μL, ANC < 1000/μL) or bendamustine-related non-hematologic toxicity that prevented starting of the next cycle within 8 days. DLT evaluation was performed only during cycle 1. If AEs equivalent to DLT were observed even outside the evaluation period, the decision of DLT was left to the Data and Safety Monitoring Board (DSMB).

### Patients

Patients aged ≥ 20 years diagnosed with advanced solid tumors and resistant to standard therapy or no standard therapy and met the selection criteria were included in the study. The main inclusion criteria were:1) patients with an Eastern Clinical Oncology Group performance status of 0–1; 2) preserved organ and bone marrow functions (absolute neutrophil count ≥ 1500/μL, platelet count ≥ 100,000/μL, hemoglobin ≥ 9 g/dL; serum creatinine ≤ 1.5 × the upper limit of the normal range or estimated creatinine clearance ≥ 50 mL/min; total serum bilirubin ≤ 1.5 × the upper limit of the normal range, AST and ALT ≤ 3.0 × the upper limit of the normal range (≤ 5.0 × the upper limit of the normal range in the case of hepatic metastasis). The main exclusion criteria were:1) active, uncontrolled, or symptomatic central nervous system metastases; 2) interstitial pneumonia, pulmonary fibrosis, or severe emphysema; 3) a history of radiation pneumonitis or idiopathic or drug-induced pneumonitis; 4) major surgery performed or scheduled, or receiving immunotherapy, antibody therapy, or other biologic or radiation therapy within 4 weeks of enrollment.; 5) receiving cytotoxic chemotherapy or hormone therapy within 14 days of enrollment and palliative radiation therapy for pain management of bone lesions within 7 days of enrollment.

### Study drug

An LFHC formulation (orally-administered bendamustine) containing 10 or 30 mg of bendamustine (as a free base) in one capsule was administered once daily in the fasted state (fasting for 2 h before administration and 1 h after administration). The individual nominal dose at each level was calculated using the body surface area (BSA), and the nearest available dose was selected using 30 mg capsules in Cohort 1 and using a combination of 10 and 30 mg capsules in Cohorts 2 and 3.

### Study evaluation

The primary endpoint was the identification of DLT in cycle 1 and the number of patients with DLT. The MTD, RD, and recommended dosing schedules were estimated based on the primary endpoints. The secondary endpoints were safety, PK, and efficacy. Efficacy was assessed as complete response (CR), partial response (PR), stable disease (SD), progressive disease (PD), or not evaluable as an objective tumor assessment based on the Response Evaluation Criteria in Solid Tumors (RECIST) criteria (version 1.1). In addition, progression-free survival (PFS) was evaluated. If DLT occurred in one of three patients at each level in each cohort, another three patients were added to the relevant cohort/level; MTD was defined as the highest level with DLT occurring in ≤ one of six patients. The RD and recommended dosing schedule were determined by an independent DSMB based on a comprehensive evaluation.

### Pharmacokinetics

Blood samples were collected at 8 time points: immediately before administration and 0.5, 1, 1.5, 2, 4, 6, and 8 h after administration. Plasma concentrations of unchanged bendamustine were determined using liquid chromatography-tandem mass spectrometry. The individual PK parameters, maximum concentration (C_max_), time to C_max_ (t_max_), AUC up to the last detectable time point (AUC_0–last_), AUC up to infinity (AUC_0–inf_), elimination half-life (t_1/2_), oral clearance (CL/F), and apparent volume of distribution (Vd/F), were calculated by non-compartment analysis. Dose proportionality was evaluated for C_max_, AUC_0–last_, and AUC_0–inf_ on day 1 using linear regression analysis based on the actual dose per BSA. A linear regression analysis between BSA and CL/F was performed to investigate the effects of BSA. The PK of a single administration were evaluated on day 1, and those of repeated administration were evaluated on days 8 and 15 of cycle 1 in cohorts 2 and 3, respectively.

### Safety

At each observation time point, AEs were evaluated based on factors, such as subjective and objective symptoms, vital signs, laboratory tests, and general condition and graded using the NCI CTCAE (version 4.03). A causal relationship with bendamustine was assessed, and AEs, wherein a causal relationship could not be ruled out, were classified as adverse drug reactions.

### Statistical analysis

Continuous variables were summarized using the number of patients, mean, and standard deviation. Categorical variables were summarized using frequencies and percentages. PFS was estimated using the Kaplan–Meier method. All analyses were performed using SAS version 9.4.

## Results

### Patient disposition and background

In this study, 22 Japanese patients provided written informed consent, and 18 patients received treatment. No patients experienced DLT at 25 mg/m^2^/day × 7 days and 50 mg/m^2^/day × 7 days; therefore, the study progressed to 75 mg/m^2^/day × 7 days. Because DLT occurred in one patient at 75 mg/m^2^/day × 7 days, three other patients were included, and a total of six patients was evaluated. Because DLT was observed in only one of six patients, 37.5 mg/m^2^/day × 14 days and 25 mg/m^2^/day × 21 days, each with three patients, were initiated simultaneously. However, because delayed hematologic recovery was more prominent at 37.5 mg/m^2^/day × 14 days and 25 mg/m^2^/day × 21 days, no further patients were enrolled.

As all 18 enrolled patients received orally-administered bendamustine, they were included in the safety and efficacy evaluation. Among the 18 patients, 10 were male (55.6%) and 8 were female (44.4%) aged 58.8 ± 10.2 years, and all had primary solid tumors. Patient characteristics are shown in Table 1.

**Table 1:** Baseline characteristics

		Total	(%)	Cohort 1			Cohort 2	Cohort 3
				Level 1	Level 2	Level 3	Level 3	Level 3
				25 mg/m^2^ × 7 d ^a^	50 mg/m^2^ × 7 d ^a^	75 mg/m^2^ × 7 d ^a^	37.5 mg/m^2^ × 14 d ^a^	25 mg/m^2^ × 21 d ^a^
Number of patients		18		3	3	6	3	3
Sex	Male	10	(55.6)	2	0	4	2	2
	Female	8	(44.4)	1	3	2	1	1
Age	(years)	58.8 ± 10.2		57.7 ± 11.9	54.7 ± 4.0	57.8 ± 14.7	62.3 ± 7.8	62.3 ± 7.4
Target advanced solid tumor	Primary	18	(100)	3	3	6	3	3
	Secondary	0	-	0	0	0	0	0
ECOG Performance status	0	9	(50.0)	0	1	5	1	2
	1	9	(50.0)	3	2	1	2	1
Prior cancer-related therapy^b^	No	0	-	0	0	0	0	0
	Yes	18	(100)	3	3	6	3	3
Number of prior cancer-related therapies	2	1	(5.6)	0	0	0	0	1
	≥ 3	17	(94.4)	3	3	6	3	2

Age is presented as mean ± standard deviation; ECOG, Eastern Cooperative Oncology Group

^a^ Daily dose is shown as mg/m^2^, and administration period is shown as d (days)

^b^ Treatment for recurrent, relapsed, or refractory advanced solid tumors prior to enrollment in this study

The number of treatment cycles for orally-administered bendamustine was 2–5 cycles at 25 mg/m^2^/day × 7 days, 1–3 cycles at 50 mg/m^2^/day × 7 days. At 75 mg/m^2^/day × 7 days, three of six patients had longer treatment periods as they underwent 7, 9, and 16 cycles, respectively. At 37.5 mg/m^2^/day × 14 days, three patients underwent 1–4 cycles, and at 25 mg/m^2^/day × 21 days, all patients underwent 2 cycles.

### Dose-limiting toxicity and maximum tolerated dose

None of the patients at 25 mg/m^2^/day × 7 days and 50 mg/m^2^/day × 7 days developed DLT. 75 mg/m^2^ × 7 days (total 525 mg/m^2^/cycle), DLT (grade 3 platelet count decreased with delay in starting the next cycle) was observed in one of six patients. 75 mg/m^2^/day × 7 days was concluded to be tolerable; however, the dose level was not escalated to 100 mg/m^2^/day × 7 days or higher due to the delayed hematologic recovery. At 75 mg/m^2^/day × 7 days, five of six patients experienced at least one delay in starting the treatment at a subsequent cycle, except for one patient who discontinued treatment due to PD at cycle 2. In addition, all patients who continued treatment from cycle 3 onward required dose reduction at cycles 3 and 5.

DLT occurred in one of three patients at 37.5 mg/m^2^/day × 14 days (Grade 3 platelet count decreased with delay in starting the next cycle) and in one of three patients at 25 mg/m^2^/day × 21 days (Grade 3 FN). The delayed hematologic recovery was more prominent at 37.5 mg/m^2^/day × 14 days, and the nadir of neutrophil and platelet counts tended to be lower, especially after repeated cycles. Consequently, the MTD was concluded to be 75 mg/m^2^ × 7 days, and a treatment schedule of once daily for 7 days every 3 weeks is recommended to allow acceptable hematologic recovery.

### Safety

Treatment-emergent AEs according to grade in each cohort (two or more appearances in all patients) are shown in Table 2. No AEs resulted in death or discontinuation of orally-administered bendamustine. Serious AEs included sepsis, *Pneumocystis jirovecii* pneumonia, and cytomegalovirus infection (n = 1 for each) at 25 mg/m^2^/day × 7 days; decreased platelet count (n = 1) at 50 mg/m^2^/day × 7 days; pneumonia (n = 1) at 75 mg/m^2^/day × 7 days; and white blood cell count decrease, cytomegalovirus infection, platelet count decrease, and FN (n = 1 for each) at 25 mg/m^2^/day × 21 days. For all serious AEs, a causal relationship with orally-administered bendamustine could not be ruled out.

**Table 2:** Adverse events by Grade in each Cohort/Level (2 or more appearances in total patients)

Event term (SOC/PT)		Total (n = 18)				Cohort 1												Cohort 2				Cohort 3			
						Level 1				Level 2				Level 3				Level 3				Level 3			
						25 mg/m^2^ × 7 d* (n = 3)				50 mg/m^2^ × 7 d* (n = 3)				75 mg/m^2^ × 7 d* (n = 6)				37.5 mg/m^2^ × 14 d* (n = 3)				25 mg/m^2^ × 21 d* (n = 3)			
Grade		1	2	3	4	1	2	3	4	1	2	3	4	1	2	3	4	1	2	3	4	1	2	3	4
Number of patients who experienced at least one event		17				2				3				6				3				3			
Blood and lymphatic system disorders																									
	Anemia	2	2	4	0	0	0	1	0	0	0	1	0	1	1	1	0	0	0	0	0	1	1	1	0
Gastrointestinal disorders																									
	Constipation	3	1	0	0	0	1	0	0	2	0	0	0	0	0	0	0	1	0	0	0	0	0	0	0
	Diarrhea	4	1	0	0	0	0	0	0	0	1	0	0	2	0	0	0	1	0	0	0	1	0	0	0
	Nausea	7	4	1	0	1	0	0	0	1	0	1	0	4	2	0	0	1	1	0	0	0	1	0	0
	Stomatitis	2	0	0	0	1	0	0	0	0	0	0	0	1	0	0	0	0	0	0	0	0	0	0	0
	Vomiting	7	3	0	0	1	0	0	0	2	0	0	0	3	2	0	0	1	1	0	0	0	0	0	0
General disorders and administration site conditions																									
	Fatigue	4	0	0	0	1	0	0	0	1	0	0	0	2	0	0	0	0	0	0	0	0	0	0	0
	Malaise	5	0	0	0	0	0	0	0	1	0	0	0	2	0	0	0	0	0	0	0	2	0	0	0
	Pyrexia	5	2	1	0	0	0	1	0	1	0	0	0	2	0	0	0	1	1	0	0	1	1	0	0
Infections and infestations																									
	Cytomegalovirus infection	0	0	2	0	0	0	1	0	0	0	0	0	0	0	0	0	0	0	0	0	0	0	1	0
	Nasopharyngitis	2	0	0	0	0	0	0	0	0	0	0	0	2	0	0	0	0	0	0	0	0	0	0	0
Investigations																									
	Alanine aminotransferase increased	5	0	0	0	0	0	0	0	1	0	0	0	1	0	0	0	2	0	0	0	1	0	0	0
	Aspartate aminotransferase increased	4	0	0	0	0	0	0	0	1	0	0	0	1	0	0	0	1	0	0	0	1	0	0	0
	Blood creatinine increased	3	1	0	0	0	0	0	0	0	0	0	0	2	0	0	0	1	1	0	0	0	0	0	0
	Blood immunoglobulin G decreased	2	0	0	0	0	0	0	0	0	0	0	0	1	0	0	0	1	0	0	0	0	0	0	0
	CD4 lymphocytes decreased	0	0	1	5	0	0	0	1	0	0	1	1	0	0	0	3	0	0	0	0	0	0	0	0
	Gamma-glutamyl transferase increased	1	1	0	0	0	0	0	0	0	0	0	0	0	0	0	0	1	0	0	0	0	1	0	0
	Lymphocyte count decreased	0	0	0	15	0	0	0	1	0	0	0	3	0	0	0	5	0	0	0	3	0	0	0	3
	Neutrophil count decreased	0	1	6	4	0	0	0	0	0	0	0	1	0	0	4	1	0	0	1	1	0	1	1	1
	Platelet count decreased	2	5	4	2	0	0	1	0	0	0	0	1	1	4	1	0	0	0	2	0	1	1	0	1
	Weight decreased	3	1	0	0	0	0	0	0	0	0	0	0	1	1	0	0	2	0	0	0	0	0	0	0
	White blood cell count decreased	0	4	10	1	0	0	1	0	0	1	1	0	0	3	3	0	0	0	3	0	0	0	2	1
Metabolism and nutrition disorders																									
	Hypoalbuminemia	0	2	0	0	0	0	0	0	0	0	0	0	0	1	0	0	0	0	0	0	0	1	0	0
	Hypokalemia	0	1	1	0	0	0	0	0	0	0	1	0	0	0	0	0	0	0	0	0	0	1	0	0
	Hypoproteinemia	2	0	0	0	0	0	0	0	1	0	0	0	0	0	0	0	0	0	0	0	1	0	0	0
	Decreased appetite	4	3	1	0	2	0	0	0	0	1	0	0	1	1	0	0	1	1	0	0	0	0	1	0
Nervous system disorders																									
	Headache	2	0	0	0	0	0	0	0	0	0	0	0	1	0	0	0	0	0	0	0	1	0	0	0
Renal and urinary disorders																									
	Renal impairment	0	2	0	0	0	0	0	0	0	0	0	0	0	1	0	0	0	1	0	0	0	0	0	0
Respiratory, thoracic and mediastinal disorders																									
	Cough	2	0	0	0	1	0	0	0	0	0	0	0	1	0	0	0	0	0	0	0	0	0	0	0
Skin and subcutaneous tissue disorders																									
	Dry skin	2	0	0	0	0	0	0	0	0	0	0	0	1	0	0	0	0	0	0	0	1	0	0	0
	Rash	4	1	0	0	0	0	0	0	1	0	0	0	2	0	0	0	0	1	0	0	1	0	0	0

The event term is shown by the Preferred term (PT) of MedDRA Ver. 22.1; the event was tabulated as the worst grade (5 > 4 > 3 > 2 > 1 > unknown); there were no events corresponding to Grade 5 in all Cohort/Level; SOC, system organ class; n, number of patients

^*^ Daily dose shown as mg/m^2^, and administration period is shown as d (days)

The DLTs observed were hematologic toxicities, such as platelet count decrease and FN, and most gastrointestinal disorders were grade 1 or grade 2, except in one patient who experienced Grade 3 nausea at 50 mg/m^2^/day × 7 days. AEs observed in this study were consistent with the known safety profiles of bendamustine injection.

### Efficacy

**Fig. 2:**
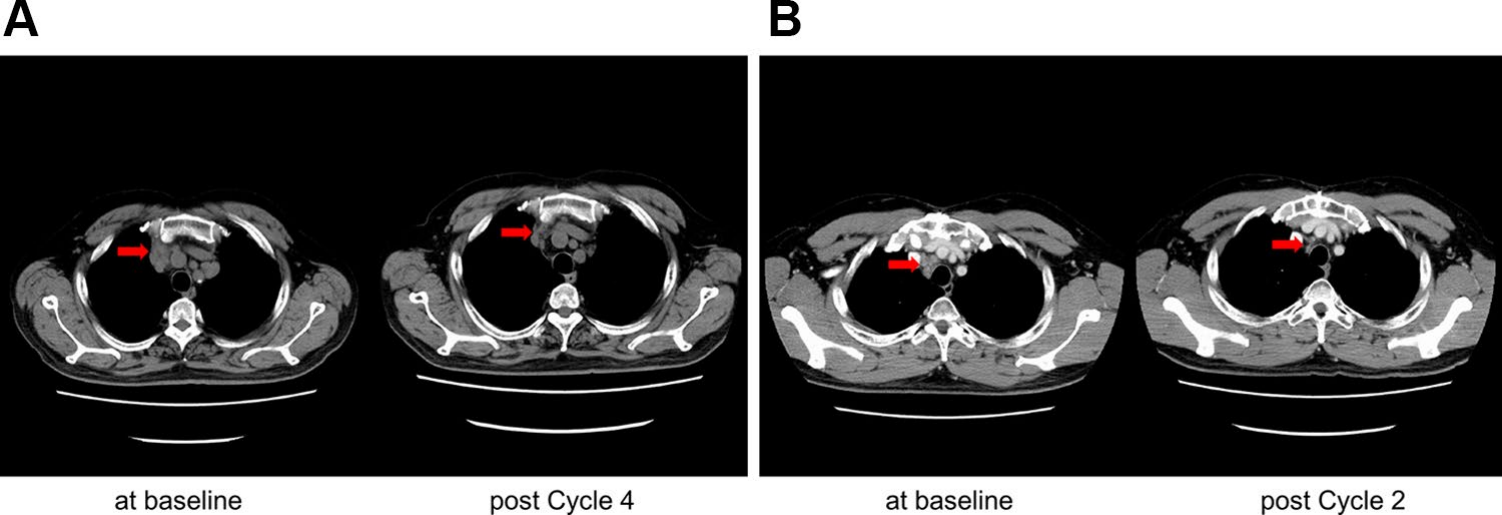
CT imaging in patients who experienced partial response. **A** S07 Prostatic small cell carcinoma treated with 75 mg/m^2^/day for 7 days and 14 days rest schedule; **B** S11 Thymic carcinoma treated with 75 mg/m^2^/day for 7 days and 14 days rest schedule; Tumor shrinkage was observed by a red arrow in both patients

**Fig. 3:**
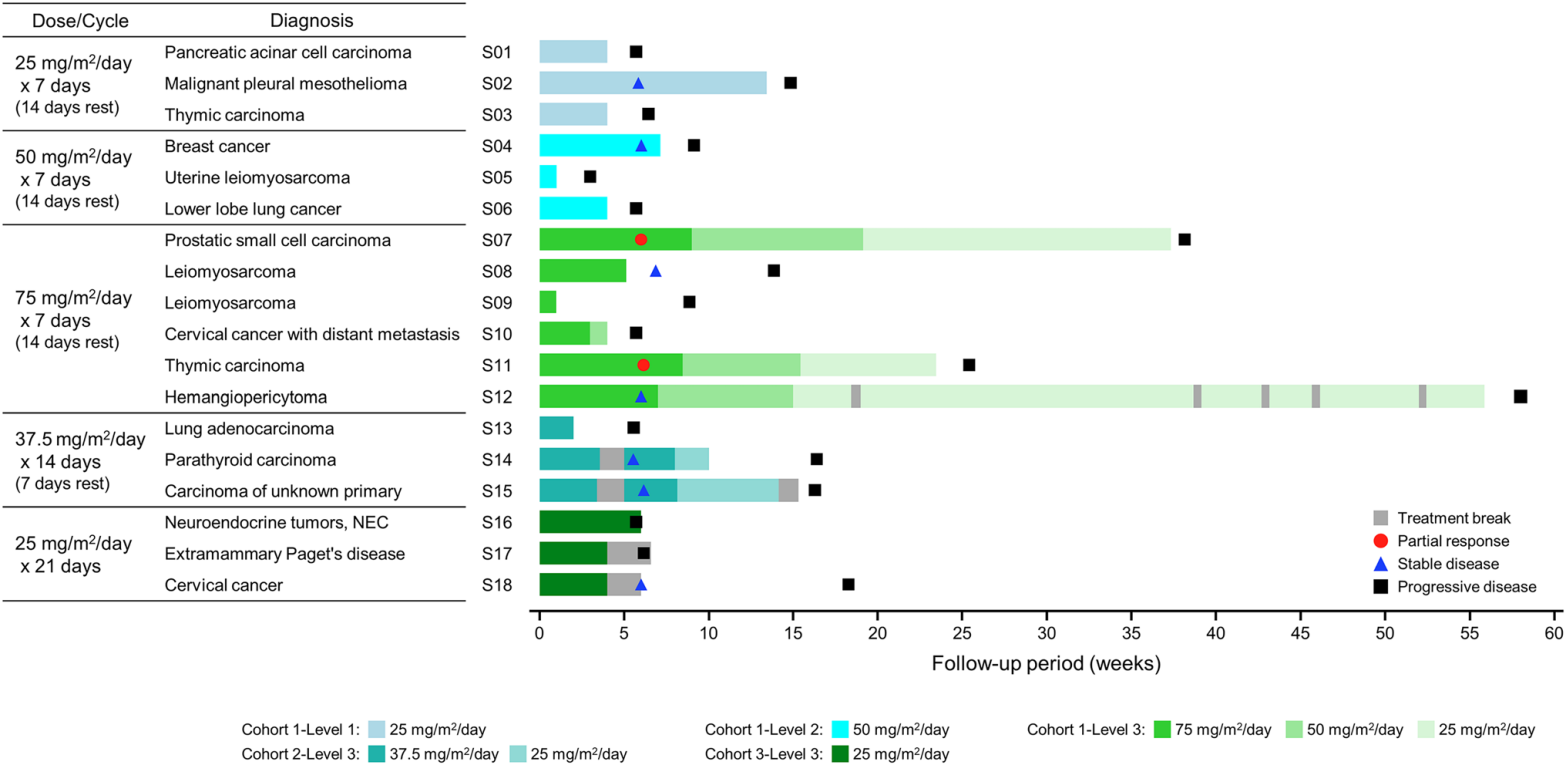
Findings for each patient (swimmer plot). *The colors in the bar chart indicate dose reductions of bendamustine

As the best overall response, no patients were assessed as having CR; however, PR was observed in two patients at 75 mg/m^2^/day × 7 days, as shown in Table S1. The overall response rate (ORR) at 75 mg/m^2^/day × 7 days was 33.3% (2/6 patients). The cancer types evaluated as PR were prostatic small cell carcinoma and thymic carcinoma in one patient each, according to computed tomography (CT) (Fig. 2). The clinical benefit rate, defined as CR, PR, or SD persisting for ≥ 16 weeks from the start of cycle 1, was 0.0% (0/3 patients) at both 25 mg/m^2^/day × 7 days and 50 mg/m^2^/day × 7 days, 50.0% (3/6 patients) at 75 mg/m^2^/day × 7 days, 66.7% (2/3 patients) at 37.5 mg/m^2^/day × 14 days, and 33.3% (1/3 patients) at 25 mg/m^2^/day × 21 days. The treatment duration for each patient is shown in a swimmer plot (Fig. 3). The PFS of each patient is shown in Table S1. The median PFS was 46.0, 41.0, 138.5, 115.0, and 44.0 days for 25 mg/m^2^/day × 7 days, 50 mg/m^2^/day × 7 days, 75 mg/m^2^/day × 7 days, 37.5 mg/m^2^/day × 14 days, and 25 mg/m^2^/day × 21 days, respectively.

### Pharmacokinetics

**Fig. 4:**
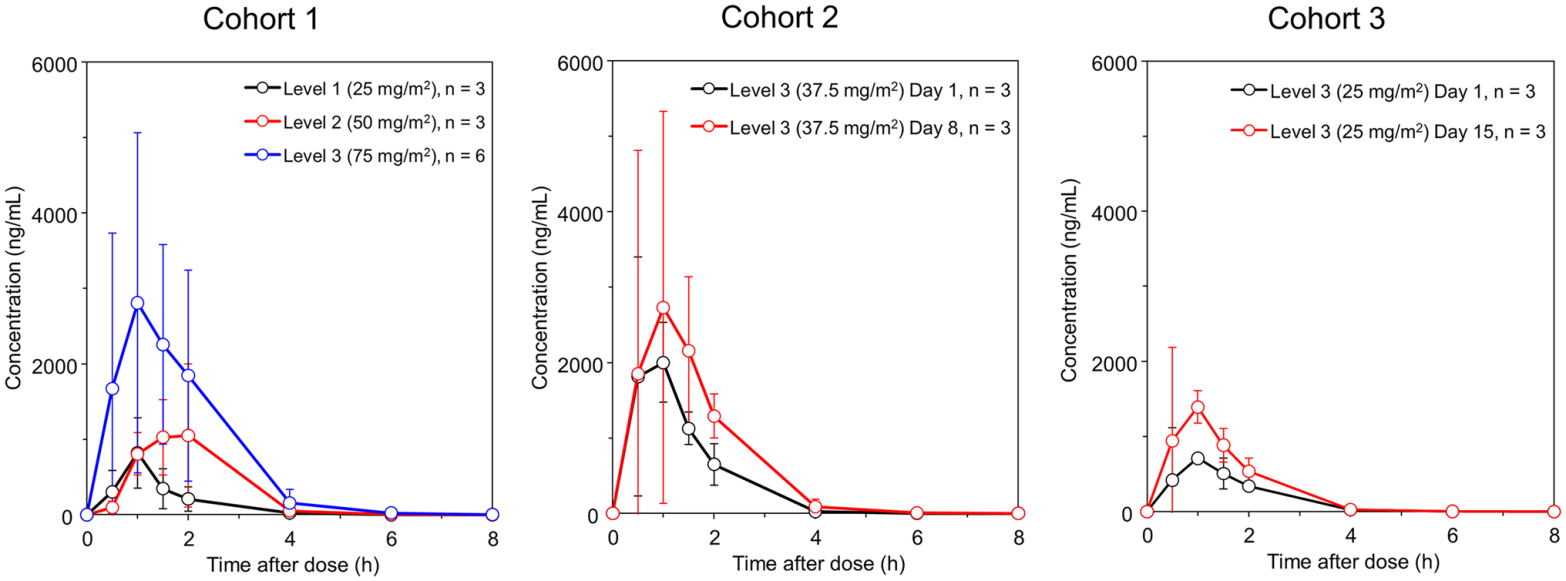
Mean concentration–time profiles of bendamustine in plasma

PK parameters were calculated for all 18 patients who received orally-administered bendamustine. The C_max_, AUC_0–last_, and AUC_0–inf_ of unchanged bendamustine on day 1 tended to increase with increasing dose. In contrast, the ranges of the mean t_max_ and t_1/2_ were not substantially different between doses (Table 3 and Fig. 4). C_max_, AUC_0–last_, and AUC_0–inf_ after repeated administration tended to increase compared with those after the first dose; however, the differences were not conclusive, owing to the large inter-individual variability and limited number of patients evaluated. Using the PK parameters on day 1, a linear regression analysis between the actual dose per unit BSA and C_max_, AUC_0–last_, and AUC_0–inf_ of unchanged bendamustine is shown in Fig. S1. The coefficients of determination were low (0.4327, 0.5911, and 0.5912) because of the relatively large inter-individual variability, and the dose proportionality was not clear. Similarly, a regression analysis between BSA and CL/F of unchanged bendamustine using the PK parameters on day 1 is shown in Fig. 3. There was no correlation between BSA and CL/F of unchanged bendamustine, suggesting that dose adjustment based on BSA did not contribute to the reduction in inter-individual variability in the bendamustine AUC.

## Discussion

After administering bendamustine orally to 18 patients with advanced solid tumors, FN (n = 1) and a decrease in platelet count (n = 2) were observed as DLT; however, no AEs led to death or discontinuation of orally-administered bendamustine. PR was obtained in one patient each with prostatic small cell carcinoma and thymic carcinoma. As two of six patients had PR at 75 mg/m^2^/day × 7 days and delayed hematologic recovery was more prominent at 37.5 mg/m2/day × 14 days and 25 mg/m2/day × 21 days, 75 mg/m^2^/day × 7 days was determined as the RD (total dose, 525 mg/m^2^/cycle), and a treatment schedule of once daily for 7 days every 3 weeks was recommended for orally-administered bendamustine for treating solid tumors. As sustained hematologic toxicity was observed, a longer recovery period was recommended for continuous treatment, which is similar to bendamustine injection.

As shown in Table S2, the bioavailability of orally-administered bendamustine was sufficiently high, and the individual variability in PK parameters was generally similar to that of bendamustine injection. The t_1/2_ after oral administration of bendamustine was 0.71 h, which was slightly longer than after intravenous administration (0.47 h); however, t_max_ was approximately 1 h, and the plasma concentration profiles over time were similar for oral and intravenous administration. Based on the PK parameters obtained following the first dose (75 mg/m^2^/day), the AUC of bendamustine over the cycle was estimated to be 42,288 ng‧h/mL, which was approximately twice the exposure following intravenous administration (AUC over cycle: 20,424 ng‧h/mL) at the maximum approved dose of intravenous bendamustine (120 mg/m^2^ once daily for 2 days every 3 weeks). Notably, regardless of the high exposure to bendamustine, gastrointestinal toxicity was not dose-limiting, and the safety profile of orally-administered bendamustine was similar to that of intravenous administration. Therefore, orally-administered bendamustine might be an alternative to intravenous bendamustine for the treatment of solid tumors and malignant lymphoma.

Bendamustine-based regimens have been recommended in the NCCN guidelines [18, 19] as first-line or second-line therapies for the treatment of follicular lymphoma, MCL, CLL, or small lymphocytic lymphoma based on pivotal phase III clinical trials [20–27]. Typically, bendamustine in combination with rituximab has been categorized as a less aggressive therapy, however, its clinical activity has been established and widely used for the treatment of malignant lymphoma. Interestingly, bendamustine is also listed in the NCCN guidelines [28] for the treatment of second-line SCLC [15], and its clinical activity has been evident in mBC [11–13]. In our clinical trial, tumor responses were observed in patients with prostatic small cell carcinoma and thymic carcinoma. These patients, S07 and S11, received 5 and 1 prior systemic therapies, respectively, and both patients received carboplatin-based prior chemotherapies and radiation therapy in common. The tumor response was observed at the first assessment in cycle 2 for both patients. Bulky lymphadenopathy and a high prevalence of metastatic disease are characteristics of these diseases [29, 30]; these are also common in SCLC [31] and mBC [32]. Although the data available in our trial are limited, bendamustine may provide a unique strategy for the treatment of lymphatic system-mediated metastatic solid tumors.

Bendamustine is rapidly eliminated from the blood, and mono-hydroxylated and di-hydroxylated bendamustine are primarily formed by rapid hydrolysis. A portion of the bendamustine is metabolized via CYP1A2 into demethylated bendamustine. Therefore, drug interactions with CYP1A2 inhibitors or inducers are unlikely because of the limited contribution of CYP1A2 to bendamustine metabolism. The effect of bendamustine exposure is not dependent on age, ethnicity, sex, or differences in liver and kidney function [33]. The bioavailability of the LFHC formulation was sufficiently high as confirmed in our study. Furthermore, as we found no correlation between the CL/F of bendamustine and BSA, a convenient flat dose may be applicable for oral administration. These PK profiles of orally-administered bendamustine are suitable for the treatment of solid tumors in outpatient settings.

The MTD, RD, and recommended treatment schedule were determined to be 75 mg/m^2^ once daily for 7 days every 3 weeks, where DLT was observed in one of six patients. Sufficient exposure to bendamustine was obtained at that dose level, and PR was achieved in two of six patients with prostatic small cell carcinoma and thymic carcinoma, suggesting the antitumor activity of orally-administered bendamustine for treating advanced solid tumors. The optimal dosing schedule for orally-administered bendamustine in patients with advanced solid tumors may be a short-term high-dose regimen with a sufficient washout period, similar to bendamustine injection in patients with hematologic malignancies. The results of our study warrant further investigation of the efficacy and safety of orally-administered bendamustine as a new treatment option for solid tumors, or possibly a new alternative to bendamustine injection for the treatment of malignant lymphoma.

## Supplementary Information

Below is the link to the electronic supplementary material.

Supplementary file1 (DOCX 56 KB)

## Data Availability

The datasets generated and analyzed during the current study are not publicly available to preserve patient confidentiality; however, these will be made available by the corresponding author upon reasonable request.
