# Supplementary material for: Oral formulation of bendamustine hydrochloride for patients with advanced solid tumors; a phase 1 study
Source: Invest New Drugs. 2022 Nov 4;41(1):1–12. doi: 10.1007/s10637-022-01307-6 (PMC10030450; doi:10.1007/s10637-022-01307-6)
Supplement: Supplementary file 1 — Supplementary file1 (DOCX 56 KB) [file 10637_2022_1307_MOESM1_ESM.docx]

**Oral formulation of bendamustine hydrochloride for patients with advanced solid tumors; a phase 1 study**

Toshio Shimizu^1,2^, Kazuhiko Nakagawa^3^, Hidetoshi Hayashi^3^, Tsutomu Iwasa^3^, Hisato Kawakami^3^, Satomi Watanabe^3^, Noboru Yamamoto^1^, Kan Yonemori^1^, Takafumi Koyama^1^, Jun Sato^1^, Kenji Tamura^1,4^, Keiichi Kikuchi^5^, Kenichiro Akaike^5^, Shiho Takeda^5^, Masayuki Takeda^3,6^

^1^ Department of Experimental Therapeutics, National Cancer Center Hospital, 5-1-1 Tsukiji Chuo-ku, Tokyo, Japan

^2^(Current institution) Department of Pulmonary Medicine and Medical Oncology, Wakayama Medical University Graduate School of Medicine, Wakayama Medical University Hospital, 811-1 Kimiidera, Wakayama-City, Wakayama, Japan

^3^Department of Medical Oncology, Kindai University Faculty of Medicine, 377-2, Osakasayama-city, Osaka, Japan

^4^Innovative Cancer Center / Department of Medical Oncology, Faculty of Medicine, Shimane University, 89-1. Enyacho Izumo-city, Shimane, Japan

^5^SymBio Pharmaceuticals Limited, 3-2-2 Toranomon, Minato-ku, Tokyo, Japan

^6^Department of Cancer Genomics and Medical Oncology, Nara Medical University, 840 Shijo-Cho, Kashihara, Nara, Japan

**Corresponding author:**

Toshio Shimizu, M.D., Ph.D.

Department of Pulmonary Medicine and Medical Oncology

Wakayama Medical University Hospital
811-1 Kimiidera, Wakayama-City
Wakayama, Japan
TEL：+81-74-347-2300
E-mail：sentants@wakayama-med.ac.jp

**Supplementary Data**

**Supplementary Table S1. Overall response and PFS**

| Cohort-Level  (Dose/cycle)* | Patient ID | Diagnosis | Overall response | PFS (day) |
| --- | --- | --- | --- | --- |
| Cohort 1-Level 1  (175 mg/m^2^; 25 mg/m2/day x 7 day) | S01 | Pancreatic acinar cell carcinoma | PD | 41 |
|  | S02 | Malignant pleural mesothelioma | SD | 105 |
|  | S03 | Thymic carcinoma | PD | 46 |
| Cohort 1-Level 2  (350 mg/m^2^; 50 mg/m^2^/day x 7 day) | S04 | Breast cancer | SD | 65 |
|  | S05 | Uterine leiomyosarcoma | PD | 22 |
|  | S06 | Lower lobe lung cancer | PD | 41 |
| Cohort 1-Level 3  (525 mg/m^2^; 75 mg/m^2^/day x 7 day) | S07 | Prostatic small cell carcinoma | PR | 268 |
|  | S08 | Leiomyosarcoma | SD | 98 |
|  | S09 | Leiomyosarcoma | PD | 63 |
|  | S10 | Cervical cancer with distant metastasis | PD | 41 |
|  | S11 | Thymic carcinoma | PR | 179 |
|  | S12 | Hemangiopericytoma | SD | 407 |
| Cohort 2-Level 3  (525 mg/m^2^; 37.5 mg/m^2^/day x 14 day) | S13 | Lung adenocarcinoma | PD | 40 |
|  | S14 | Parathyroid carcinoma | SD | 116 |
|  | S15 | Carcinoma of unknown primary | SD | 115 |
| Cohort 3-Level 3  (525 mg/m^2^; 25 mg/m^2^/day x 21 day) | S16 | Neuroendocrine tumors, NEC | PD | 41 |
|  | S17 | Extramammary Paget's disease | PD | 44 |
|  | S18 | Cervical cancer | SD | 129 |

PFS, progression-free survival; PD, progressive disease; SD, stable disease; PR, partial response.

*Total dose of bendamustine in 1 cycle (3-week schedule).

**Supplementary Table S2. Comparison of pharmacokinetic parameters of bendamustine**

| PO administration | Dose/day |  | C_max_ (ng/mL) | t_max_ (h) | AUC_0–last_ (ng⸱h/mL) | t_1/2_ (h) | Vd/F (mL) | CL/F (mL/h) | AUC/cycle (ng⸱h/mL) |
| --- | --- | --- | --- | --- | --- | --- | --- | --- | --- |
| PO Cohort 1-Level 3 (1-week dose/2-week rest) | 75 mg/m^2^ (as free base) | n | 6 | 6 | 6 | 6 | 6 | 6 |  |
|  |  | Mean | 3642 | 1.42 | 6041 | 0.71 | 31244 | 29693 | 42288 |
|  |  | SD | 1952 | 0.66 | 2611 | 0.12 | 23261 | 19665 |  |
| IV administration^a^ | Dose/day |  | C_max_ (ng/mL) | t_max_ (h) | AUC_0–t_ (ng⸱h/mL) | t_1/2_ (h) | Vz (mL) | CL (mL/h) | AUC/cycle (ng⸱h/mL) |
| IV 1 h infusion for 2 consecutive days | 120 mg/m^2^ (109 mg/m^2^ as free base) | n | 6 | 6 | 6 | 6 | 6 | 6 |  |
|  |  | Mean | 8616 | 0.90 | 10212 | 0.47 | 17532 | 25963 | 20424 |
|  |  | SD | 4488 | 0.20 | 5759 | 0.05 | 10578 | 15531 |  |

PO, per OS; IV, intravenous injection; C_max_, maximum plasma drug concentration; t_max_, time to C_max_; AUC_0–last_, area under the plasma concentration-time curve until the last plasma concentration detection time; t_1/2_, elimination half-life; Vd/F, apparent volume of distribution; Vz, volume of distribution calculated from elimination phase; CL/F, oral clearance; CL, total body clearance; AUC/cycle, area under the plasma concentration-time curve per cycle; n, number of patients; SD, standard deviation.

^a^ Package insert in Japan, TREAKISYM^®^ injection solution.

**Supplementary Figure S1. Correlation between actual dose per BSA and pharmacokinetic parameters at Day1.**

C_max_, maximum plasma drug concentration; AUC_0–last_, area under the plasma concentration-time curve until the last plasma concentration detection time; AUC_0–inf_, area under the plasma concentration-time curve until infinity; BSA, body surface area

**Supplementary Figure S2. Correlations between BSA and oral clearance at Day 1**

CL/F, oral clearance; BSA, body surface area
